# Post-traumatic growth and rehabilitation adherence in lower extremity fracture patients: a parallel mediation model

**DOI:** 10.3389/fpsyg.2026.1773501

**Published:** 2026-01-30

**Authors:** Xiaowei Liu, Qianwen Chen, Danqin Li, Huixiu Xiong, Han Shi, Xuanying Li, Yujie He, Xiaoxiao Mei, Zengjie Ye

**Affiliations:** 1 School of Nursing, Guangzhou University of Chinese Medicine, Guangzhou, Guangdong, China; 2 Foshan Hospital of Traditional Chinese Medicine, Foshan, Guangdong, China; 3 School of Nursing, The Hong Kong Polytechnic University, Hong Kong, Hong Kong SAR, China; 4 School of Nursing, Guangzhou Medical University, Guangzhou, Guangdong, China

**Keywords:** adherence to rehabilitation exercises, fracture, perceived social support, post-traumatic growth, self-efficacy

## Abstract

**Background:**

Previous studies have shown that psychological factors such as post-traumatic growth (PTG), self-efficacy, and social support may be associated with rehabilitation behaviors in patients with musculoskeletal injuries. The mechanism underlying the relationship between PTG and adherence to rehabilitation exercises among patients with lower extremity fractures needs to be further investigated.

**Methods:**

In total, 407 patients with lower extremity fractures were recruited from the Be Resilient to Fractures Cohort. Assessment tools measured post-traumatic growth, self-efficacy, perceived social support, and adherence to rehabilitation exercises. Bootstrap-based structural equation modeling was used to analyze data.

**Results:**

The structural equation model showed a good fit, with χ^2^/df = 2.879, RMSEA = 0.068, GFI = 0.933, CFI = 0.972, and AGFI = 0.900. Post-traumatic growth was positively associated with adherence to rehabilitation exercises (*β* = 0.149, 95%CI [0.014,0.289], *p* = 0.028). Both self-efficacy (*β* = 0.261, 95%CI [0.193,0.340], *p* < 0.01) and perceived social support (*β* = 0.176, 95%CI [0.087,0.271], *p* < 0.01) significantly mediated this relationship. Following adjustments for income, all paths remained significant. Measurement invariance was established across gender groups.

**Conclusion:**

The findings underscore the importance of fostering post-traumatic growth, enhancing self-efficacy, and strengthening social support systems to improve rehabilitation outcomes for patients with lower extremity fractures.

## 1 Introduction

Lower extremity fractures represent a common trauma, predominantly resulting from high-energy injuries or pathological factors (Zak et al., 2021; Fu et al., 2025). Beyond physical impairment, these injuries frequently lead to significant psychological distress (Angenendt, 2021; Maddison et al., 2023). Adherence to rehabilitation exercises (REA) is critical for recovery, yet reports suggested 50–70% of patients struggled with adherence due to pain and psychological barriers, impairing functional outcomes (Wang et al., 2025). Therefore, identifying modifiable factors that influence adherence is essential (Fernandes et al., 2023; Fernandes et al., 2024).

The Protection Motivation Theory (PMT) offers a conceptual model for analyzing health-related behavioral patterns. It posits that the intention to adopt protective behaviors, such as adherence to rehabilitation exercises (REA), is shaped by both threat appraisal and coping appraisal (Rogers, 1975). Within this framework, self-efficacy (SE) and perceived social support (PSS) are two pivotal components of coping appraisal that motivate action. SE denotes a person’s conviction in their capacity to perform particular actions (Ou, 2022). In contrast, PSS refers to the belief that supportive resources are available from one’s social network (Zhu et al., 2024). Research has demonstrated the utility of this theoretical framework in forecasting compliance with rehabilitation protocols, as evidenced by prior studies (Fu et al., 2025). It suggests that patients are more likely to adhere when they recognize prescribed exercises as a viable coping mechanism to mitigate the health risks associated with fractures.

**Figure 1:**
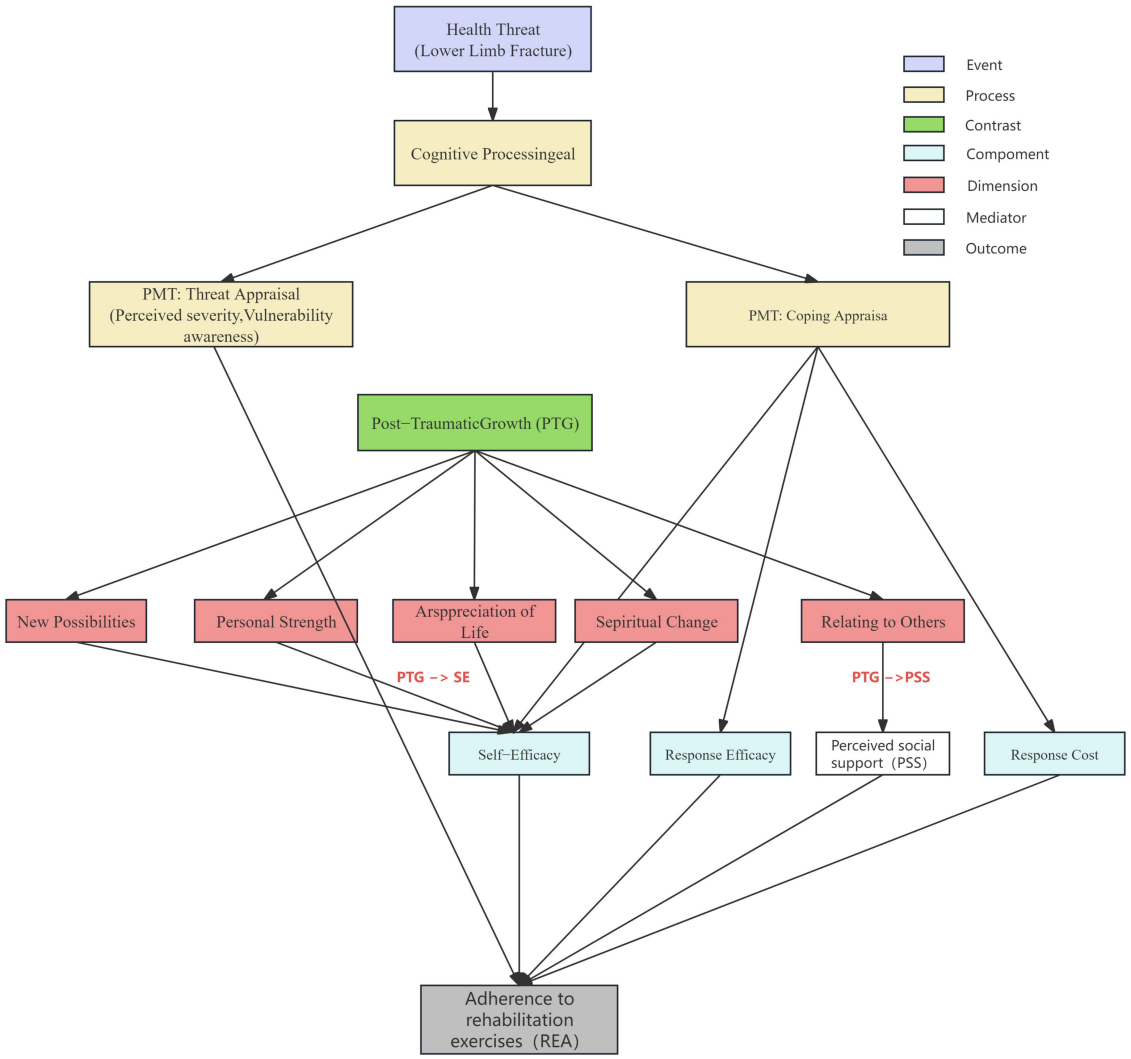
Theoretical framework linking protection motivation theory and post-traumatic growth.

Post-traumatic growth (PTG) is conceptualized as a positive psychological transformation experienced following a traumatic event (Jayawickreme et al., 2021; Magne et al., 2021). It offers a crucial lens for understanding post-fracture adaptation. PTG is linked to beneficial health behaviors (Zhang et al., 2025; Dyball et al., 2025; Kalbas et al., 2023). Critically, we propose that PTG can be integrated into the PMT framework by serving as an upstream psychological resource that strengthens the coping appraisal. Specifically, the process of achieving PTG, characterized by a revised sense of personal strength and enhanced relationships, is positively associated with increased SE and PSS, respectively (Jayawickreme et al., 2021; Jones et al., 2020). Therefore, the integration of PTG within the frameworks of PMT offers a nuanced understanding of the mechanisms underlying psychological resilience in patients recovering from lower limb fractures. Thus, the association between PTG and REA may operate through SE and PSS, thereby linking PTG to the core coping appraisal mechanism of PMT. This integrated model provides a nuanced understanding of resilience and motivation in recovery (Figure 1).

Gender as a significant sociodemographic variable may further moderate these psychological pathways, as studies indicate it influences post-trauma responses (Rønning et al., 2025; Caycho-Rodríguez et al., 2025). Therefore, gender differences should be considered in the design of mental health interventions that foster PTG. However, its role in the present context is unknown.

To address these gaps, this research delved into the relationship between Posttraumatic Growth and Rehabilitation Engagement among patients recovering from lower limb fractures, employing a comprehensive Protection Motivation Theory framework as our analytical lens. Specifically, we examine the parallel mediating roles of SE and PSS in this relationship and test whether the proposed model is invariant across genders. The following hypotheses are posited:

*H1*: PTG is positively associated with REA.

*H2*: SE mediates the relationship between PTG and REA.

*H3*: PSS mediates the relationship between PTG and REA.

*H4*: The mediation model exhibits measurement equivalence across genders.

## 2 Methods and analysis

### 2.1 Study design

As a component of the ongoing “Be Resilient to Fractures” (BRTF) Cohort study, the present research is based on a cross-sectional design. The BRTF aims to track the long-term effects of fractures on individuals. During this initial phase, a cohort of patients presenting with lower limb fractures was recruited from the orthopedic traumatology department at a tertiary hospital between December 2024 and September 2025. The findings of this study were reported in accordance with the Strengthening the Reporting of Observational Studies in Epidemiology (STROBE) Statement.

### 2.2 Sample size

**Figure 2:**
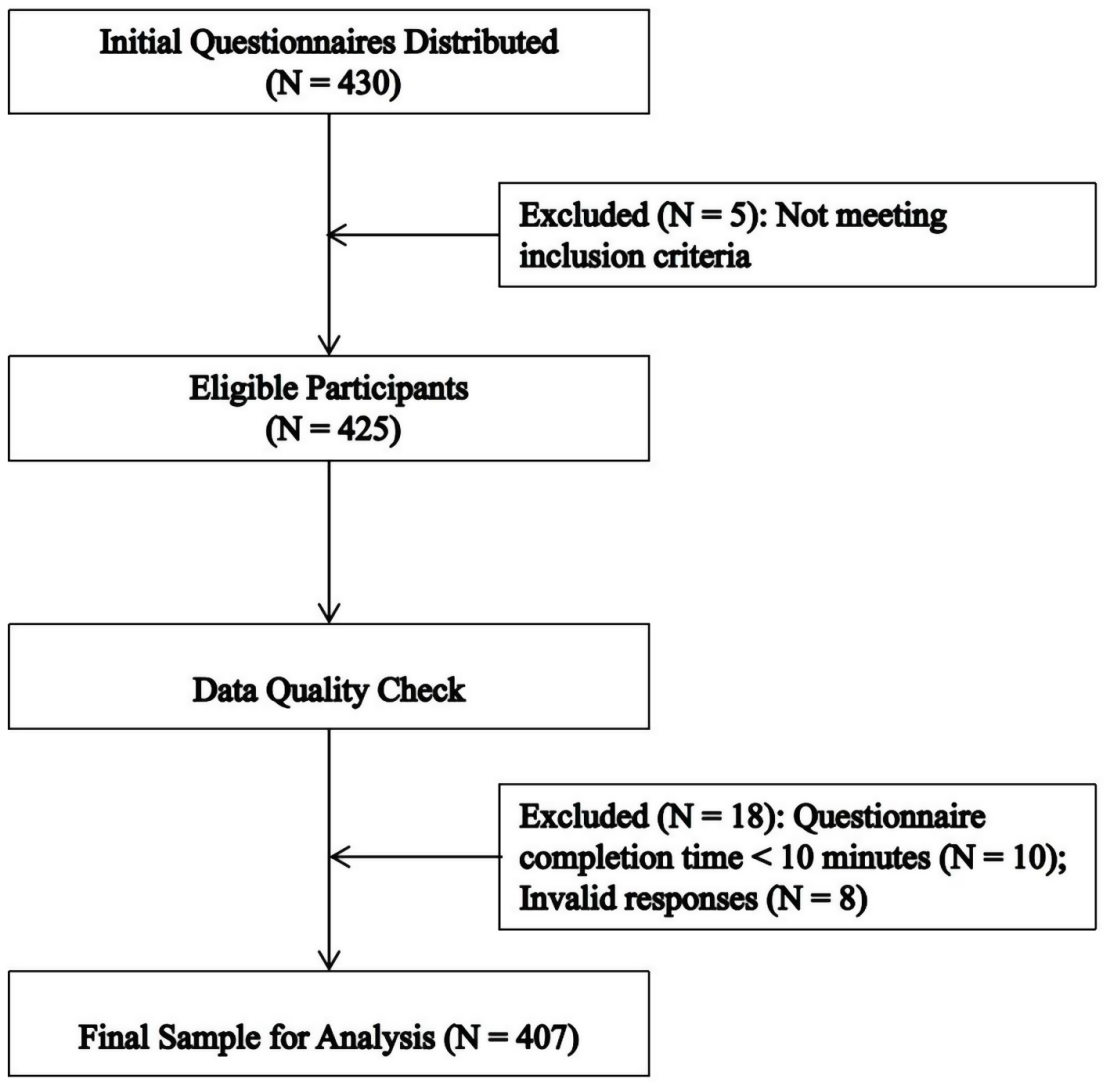
Participant flowchart.

Following the guidelines for sample size estimation in structural equation modeling, the required sample size should exceed 200 cases (Bentler and Yuan, 1999), adhering to a ratio of at least 10 participants per observed variable (Riley et al., 2020). This study included 21 variables, necessitating a sample of 252 to accommodate a 20% inefficiency in the sample size. In total, 430 questionnaires yielded 407 valid responses, a 94.65% return rate. This rate exceeded the minimum acceptable threshold. The participant flow is illustrated in Figure 2. Before initiating data gathering procedures, ethical clearance was secured from the Institutional Review Board. Participants were required to review and sign the informed consent document presented on the questionnaire’s first page. Written consent was acquired from all individuals before their inclusion in the research.

### 2.3 Inclusion and exclusion criteria

#### 2.3.1 Inclusion criteria

(1) Age ≥ 18 years;
(2) diagnosed with unilateral lower limb fracture (including hip, femur, tibia-fibula, ankle, etc.) by imaging;
(3) hospitalization days ≥3 days, and intended to be discharged from the hospital;
(4) conscious, language expression is smooth, and can correctly understand the questions asked by the investigator.

#### 2.3.2 Exclusion criteria

(1) Low intelligence or post-injury language and emotional disorders;
(2) serious physical diseases before injury, such as heart, lung, liver, kidney, and brain diseases with serious organic lesions;
(3) mental diseases and the use of antipsychotics and antidepressants.

### 2.4 Measurements

#### 2.4.1 Demographic questionnaire

Informed by previous studies (Wang et al., 2025), we collected data on socio-demographic factors, including gender, age, family economic situation, ethnicity, disease payment methods, and body mass index (BMI).

#### 2.4.2 Post-traumatic growth inventory (PTGI)

The PTGI served as our tool for assessing personal growth after trauma. We utilized the Chinese translation of this originally 20-item questionnaire, which was developed by Tedeschi and Calhoun (1996), and subsequently translated by Wang et al. (2011). It measures five dimensions: appreciation of life (6 questions), interpersonal relationships (3 questions), new possibilities (4 questions), individual strength (3 questions), and spiritual transformation (4 questions). Respondents rated each item using a 6-point Likert scale (0 = no change, 5 = substantial change). The total possible score ranges from 0 to 100, with higher scores reflecting more significant PTG. The PTGI has demonstrated good reliability and validity in populations with traumatic fractures (Chen et al., 2025). For our investigation, the internal consistency reliability (Cronbach’s *α*) of the PTGI proved to be excellent at 0.946.

#### 2.4.3 Orthopaedic patient’s adherence to functional exercise scale

Created by Tan Yuanyuan in 2019 (Tan et al., 2019), this evaluation tool consists of 15 questions measuring three key aspects: mental state (8 questions), engagement in learning (4 questions), and bodily condition (3 questions). Responses were collected through a 5-level rating system, with possible cumulative scores ranging from 15 to 75 points. The assessment device has proven dependable through verification procedures and has been previously utilized in investigations focusing on bone-related patient populations (Zhou et al., 2023). In the present research subjects, the instrument produced an internal consistency value of 0.971 as measured by Cronbach’s alpha.

#### 2.4.4 General self-efficacy scale (GSES)

In this study, the research team employed the Chinese version of GSES as a measurement tool. Originally developed by Schwarzer and his research team in 1981, this assessment instrument was subsequently modified for Chinese cultural contexts through a collaborative effort between Zhang and Schwarzer in 1995 (Zhang and Schwarzer, 1995) scale consists of 10 statements that participants rate using a four-level Likert response format, producing composite scores that vary between 10 and 40 points. Elevated scores on this measure indicate stronger self-efficacy beliefs. Previous validation studies have confirmed the scale’s psychometric soundness when used with orthopedic patient groups (Gounelle et al., 2022). Our current data analysis revealed an excellent internal consistency coefficient of 0.954 for this instrument.

#### 2.4.5 Perceived social support scale (PSSS)

The PSSS served as an assessment tool to gage individuals’ subjective evaluations regarding the accessibility of social support. This measurement instrument was initially created by Zimet et al. (1990). Comprising 12 distinct items, the questionnaire categorizes perceived assistance into three primary domains: familial support, peer support, and support from additional sources. Respondents indicate their level of agreement using a 7-point rating system. Possible cumulative scores range from 12 to 84, with higher numerical values reflecting stronger perceptions of available social support. Previous research has demonstrated the effective application of this instrument among post-operative fracture patients (Zhou et al., 2025). In this investigation, the measure exhibited outstanding internal consistency, achieving a Cronbach’s *α* of 0.963.

### 2.5 Statistical analysis

Categorical data is shown as frequency counts and proportional values, while continuous data is expressed in terms of means and standard deviations. Before conducting parametric tests, the homogeneity of variances across compared groups was verified using Levene’s test, and the results were non-significant (all *p* > 0.05), thereby satisfying the prerequisites for parametric analysis. Subsequently, independent samples *t*-tests and one-way ANOVA were employed to examine sociodemographic differences in REA. Associations among PTG, SE, PSS, and REA were assessed using Pearson’s correlation analysis.

A mediation analysis was performed utilizing AMOS 24.0 to test the hypothesized model. To account for a key potential confounder, monthly household income was incorporated as a covariate, with direct paths specified from income to the mediators (SE, PSS) and the outcome variable (REA). This allows the estimated paths among PTG, SE, PSS, and REA to be interpreted as effects above and beyond the influence of income. This analysis utilized Bootstrap sampling, incorporating 5,000 iterations and a 95% confidence interval (CI). Statistical significance of mediation was confirmed when the 95% confidence interval did not include zero, and the *p*-value was less than 0.05. The adequacy of the model fit was assessed according to the subsequent criteria: χ^2^/df < 3, RMSEA < 0.08, GFI > 0.90, CFI > 0.90, TLI > 0.90, and AGFI > 0.90 (Streiner, 2006; Bentler, 1990).

A *post hoc* power analysis for the mediation model was conducted utilizing using G*Power 3.1 (Faul et al., 2007). We used the exact test for a proportion differing from a constant (binomial test, one-sample case). The effect size ‘*g*’ was derived from the obtained standardized indirect effect estimates and their CI, with an alpha level set at 0.05 and a sample size of 407.

Multigroup analyses were conducted to test the cross-gender measurement equivalence of the proposed mediation model. Measurement equivalence was assessed sequentially across five levels: unconstrained, measurement weights, structural covariances, structural residuals, and strict (measurement residuals) equivalence. The model fit was assessed based on the following criteria: ΔCFI < 0.01, ΔRMSEA < 0.015, and a nonsignificant chi-square difference test (*p* > 0.05) (Lasker, 2024; Protzko, 2024).

## 3 Results

### 3.1 General data of all enrollments

The final sample comprised 407 patients with lower limb fractures, averaging 43.40 ± 14.12 years in age. Demographically, 64.62% were male, 17.20% met obesity criteria, and 40.05% had attained a junior high school education or less. Additionally, 63.64% of participants were married, while 52.33% inhabited rural areas. Adherence to Rehabilitation Exercises showed no significant variations across various demographic attributes, including gender, age, BMI, educational background, marital status, place of residence, or fracture site. The complete demographic details are presented in Table 1. Table 2 displays the descriptive statistics for all variables included in the study.

**Table 1:** The difference in sociodemographic characteristics in the score of adherence to functional exercise.

Demographic category	*n* (%)	REA score (mean ± SD)	*t*/*F* value	*p*-value
Gender			*t* = −0.582	0.561
Male	263 (64.62)	45.76 ± 12.23		
Female	144 (35.38)	46.5 ± 12.10		
Age (years)			*F* = 0.886	0.347
18–35	127 (31.2)	46.84 ± 13.00		
36–50	135 (33.17)	44.60 ± 11.46		
>50	145 (35.63)	46.63 ± 12.05		
BMI (kg/m^2^)			*F* = 0.780	0.459
<24 (normal)	195 (47.91)	46.74 ± 12.29		
24–28 (overweight)	142 (34.89)	45.68 ± 11.02		
>28 (obese)	70 (17.20)	44.74 ± 14.01		
Educational			*F* = 1.707	0.184
Junior high school or below	163 (40.05)	44.98 ± 11.29		
Senior high school	137 (33.66)	45.93 ± 12.93		
Bachelor or above	107 (23.29)	47.74 ± 12.41		
Marital status			*F* = 1.085	0.339
Single	148 (36.36)	46.84 ± 12.68		
Married, ≤1 child	47 (11.55)	43.85 ± 13.28		
Married, ≥2 children	212 (52.09)	45.94 ± 11.55		
Monthly income (CNY)			*F* = 3.676	0.026
<3,000	132 (32.43)	43.98 ± 11.40		
3,000–5,000	139 (34.15)	46.04 ± 11.96		
>5,000	136 (33.42)	47.99 ± 12.87		
Residence			*t* = 1.242	0.215
Urban	194 (47.67)	46.81 ± 12.46		
Rural	213 (52.33)	45.31 ± 11.89		
Fracture site			*F* = 2.321	0.099
Femur	29 (7.13)	42.03 ± 12.00		
Tibia/fibula	221 (54.30)	46.92 ± 12.18		
Ankle and below	157 (38.57)	45.50 ± 12.10		

REA, adherence to rehabilitation exercises; BMI, body mass index.

**Table 2:** Description of the variables.

Variable	*M*	SD	Min	Max	Possible range
PTG	68.34	12.30	20	100	0–100
PTG1	10.85	2.10	3	15	0–15
PTG2	9.90	2.24	3	15	0–15
PTG3	12.95	2.79	4	20	0–20
PTG4	21.37	4.08	6	30	0–30
PTG5	13.28	2.85	4	20	0–20
SE	24.75	6.92	10	40	10–40
PSS	57.58	12.84	13	84	12–84
PSS1	20.12	4.65	4	28	4–28
PSS2	18.89	4.77	4	28	4–28
PSS3	18.57	4.31	4	28	4–28
REA	46.02	12.18	15	75	15–75
REA1	25.18	6.63	8	40	8–40
REA2	12.14	3.57	4	20	4–20
REA3	8.70	2.76	3	15	3–15

PTG, post-traumatic growth; PTG1: personal strength; PTG2: relationships with others; PTG3: new possibilities; PTG4: sense of life; PTG5: self-transformation; SE: self-efficacy; PSS, perceived social support; PSS1: family support; PSS2: friend support; PSS3: social support; REA: adherence to rehabilitation exercises; REA1: psychological aspects; REA2: active learning; REA3: physical aspects.

### 3.2 Assessment of common method Bias

The potential for common method bias was assessed using Harman’s single-factor test. In this test, a single factor explained 42.9% of the total variance, which is below the commonly cited threshold of 50% (Podsakoff et al., 2003). Therefore, substantial common method bias did not unduly influence the results of the subsequent analyses.

### 3.3 Correlation analysis

**Figure 3:**
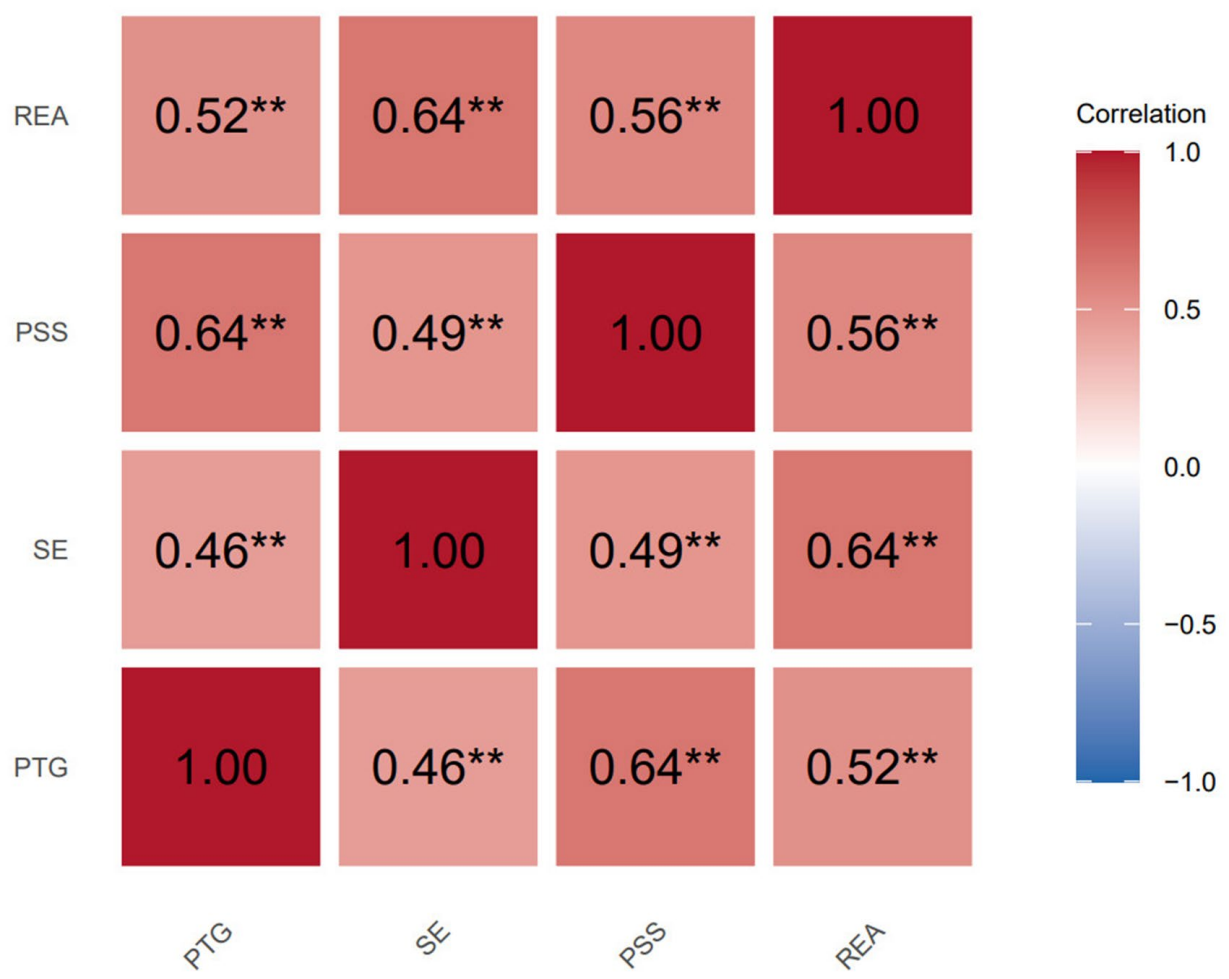
Correlation matrix. PTG, post-traumatic growth; SE, self-efficacy; PSS, perceived social support: REA, adherence to rehabilitation exercises. Values range from −1 (strong negative) to +1 (strong positive), *p* < 0.05, **p < 0.01, ***p < 0.001.

The REA in patients with lower limb fractures was positively correlated with PTG, SE, and PSS (*r* = 0.520, 0.642, and 0.556, respectively; all *p* < 0.01). PTG supported a positive association with SE and PSS (*r* = 0.456 and 0.635; both *p* < 0.01), and SE supported a positive association with PSS (*r* = 0.492, *p* < 0.01). These relationships are illustrated in Figure 3.

### 3.4 Mediation analysis

**Figure 4:**
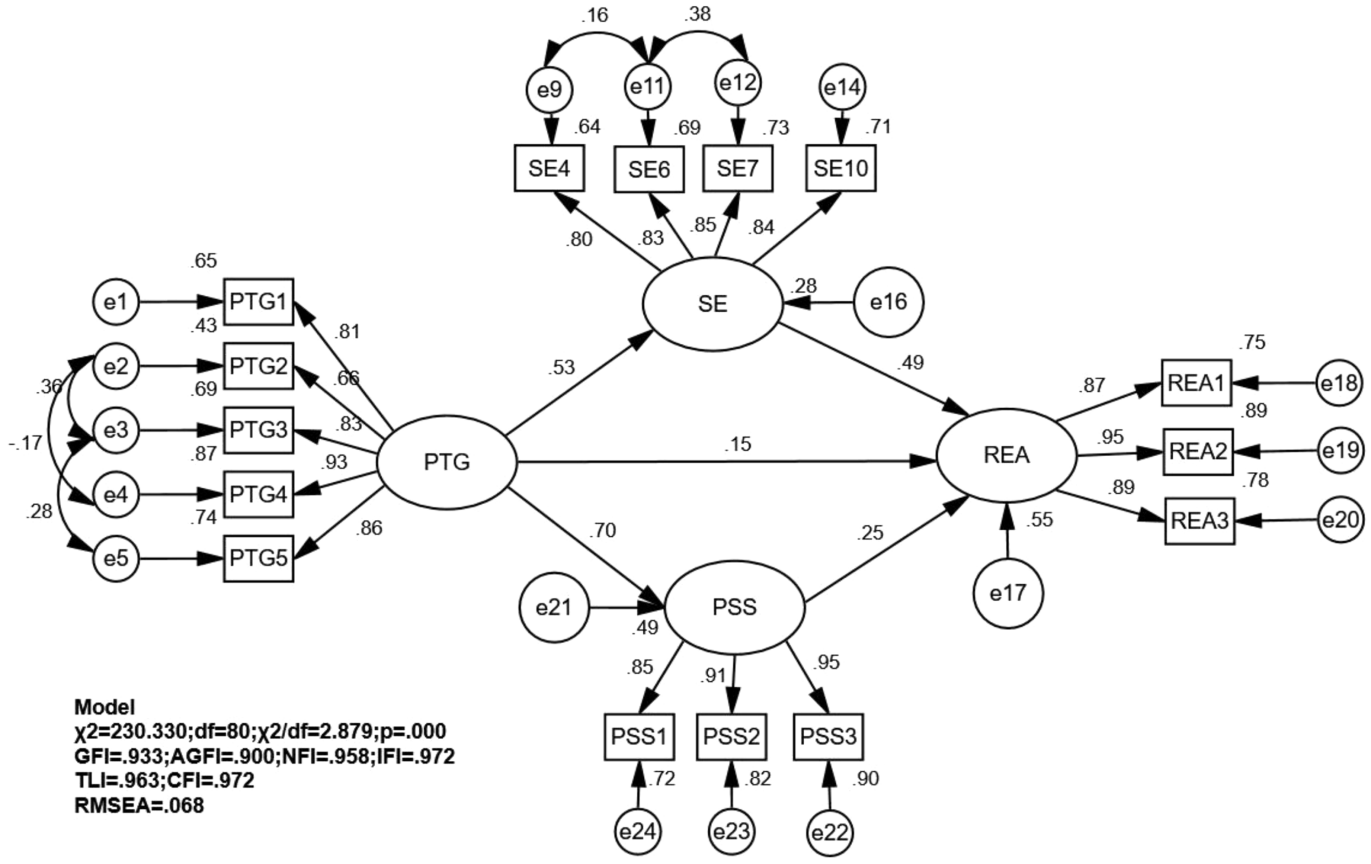
Mediation analysis of post-traumatic growth and adherence to rehabilitation exercises (unadjusted). PTGI, personal strength; PTG2, relationships with others; PTG3, new possibilities; PTG4, sense of life, PTG5, self transformation; PSSI, family support; PSS2, friend support; PSS3, social support; REAI, psychological aspects; REA2, active learning; REA3, physical aspects; SE4, I am confident in my ability to effectively manage any unexpected situations that arise; SE6, if I make the necessary efforts, I am certain to solve most of the difficult problems; SE7, I can face difficulties calmly because I trust my ability to handle problems; SE10, I am able to cope with whatever happens to me. Independent variable (X): PTG. Mediator variables (M): SE(M1), PSS(M2). Dependent variable (Y): REA. Diagram structure: Direct effect: Path PTG(X) → REA(Y). Path coefficient: *β* = 0.149, *p* < 0.05. Indirect effects via SE: PTG(X) → SE(M1) → REA(Y). Indirect effect: *β* = 0.261, 95%CI [0.193, 0.340], *p* < 0.01. Indirect effects via PSS: Path PTG(X) → PSS(M2) → REA(Y). Indirect effect: *β* = 0.176, 95% CI [0.087, 0.271], *p* < 0.01. Total effect (sum of direct and indirect effects): *β* = 0.586, 95% CI [0.495, 0.665], *p* < 0.01.

**Figure 5:**
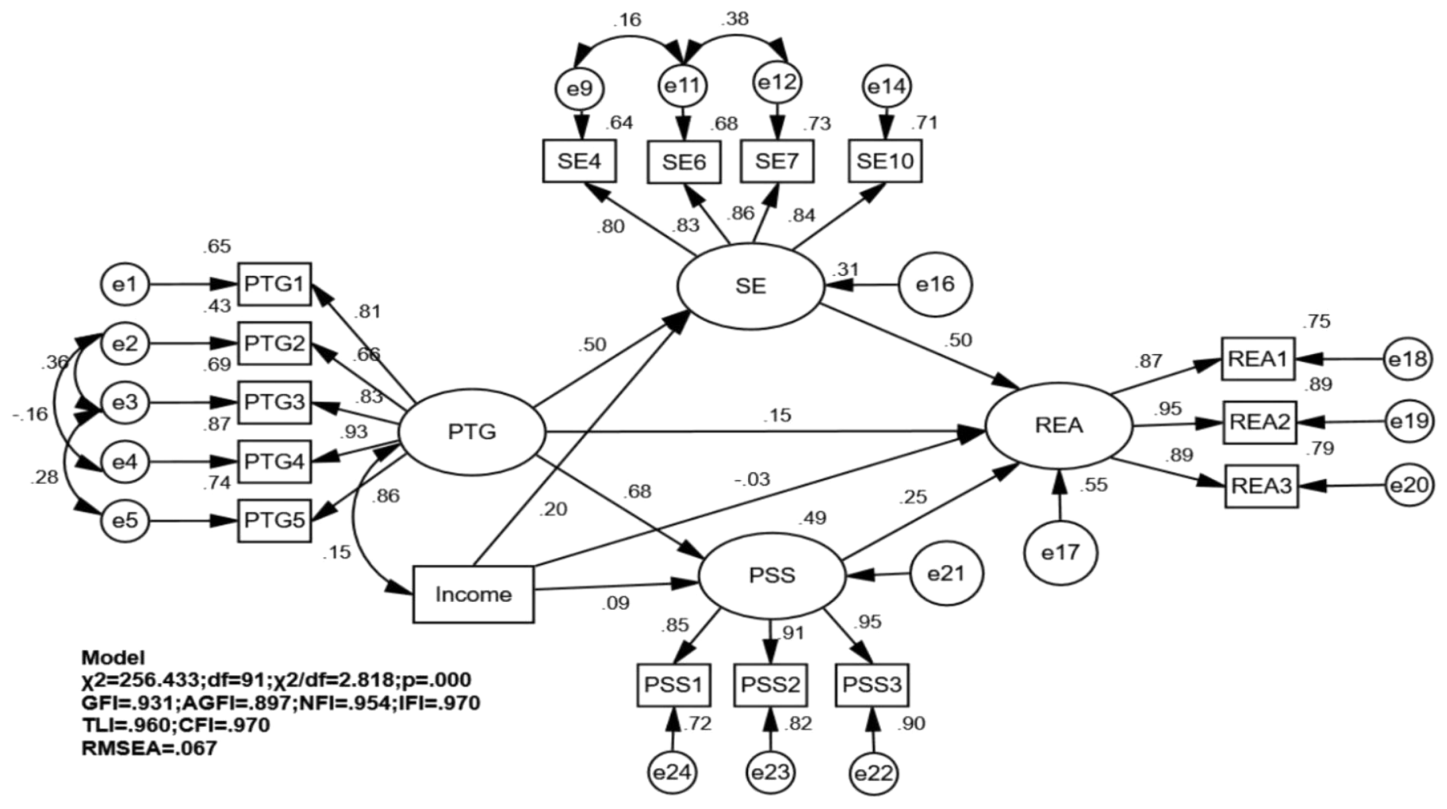
Mediation analysis of post-traumatic growth and adherence to rehabilitation exercises (adjusted gor income). PTGI, personal strength; PTG2, relationships with others; PTG3, new possibilities; PTG4, sense of life; PTG5S, self transformation; PSSI, family support; PSS2, friend support; PSS3, social support; REAI, psychological aspects; REA2, active learning; REA3, physical aspects, SE4, I am confident in my ability to effectively manage any unexpected situations that arise; SE6, if I make the necessary efforts, I am certain to solve most of the difficult problems: SE7, I can face difficulties calmly because I trust my ability to handle problems; SE10, I am able to cope with whatever happens to me. Independent variable (X): PTG. Mediator variables (M): SE(MI), PSS(M2). Dependent variable (Y): REA. Control variable: Income. Diagram structure: Direct effect: Patir PTG(X) → REA(Y). Path coefficient: *β* = 0.003149, *p* < 0.05. Indirect effects via SE: PTG(X) → SE(MI) → REA(Y). Indirect effect: *β* = 0.249, 95%CI [0.181, 0.330], *p* < 0.01. Indirect effects via PSS: Path PTG(X) → PSS(M2) → REA(Y). Indirect effect: *β* = 0.173, 95% CI [0.086, 0.264], *p* < 0.01. Total effect (Sum of direct and indirect effects): *β* = 0.571, 95% CI [0.476, 0.656], *p* < 0.01.

The revised model exhibited good fit indices: *χ*^2^ = 230.330, χ^2^/df = 2.879, RMSEA = 0.068, AGFI = 0.900, GFI = 0.933, TLI = 0.963, CFI = 0.972 and IFI = 0.972. As illustrated in Figure 4, path analysis revealed that PTG in lower limb fracture patients was positively associated with REA (*β* = 0.149, *p* < 0.05), SE (*β* = 0.528, *p* < 0.01), and PSS (*β* = 0.698, *p* < 0.01). Additionally, significant positive associations with REA were observed for SE (*β* = 0.494, *p* < 0.01) and PSS (*β* = 0.252, *p* < 0.01). Bootstrap mediation analysis indicated significant indirect associations between PTG and REA through two pathways: via SE (*β* = 0.261, 95% CI [0.193, 0.340], *p* < 0.01) and PSS (*β* = 0.176, 95% CI [0.087, 0.271], *p* < 0.01). The direct, indirect, and overall effects are thoroughly outlined in Table 3 and Figure 4. Following adjustments for income, all paths remain significant, as shown in Table 4. Although AGFI of 0.897 was slightly below 0.90, the primary fit indices (χ^2^/df = 2.818, RMSEA = 0.067, GFI = 0.931, CFI = 0.970) all met excellent criteria, supporting the overall acceptability of the model, as shown in Figure 5. The *post hoc* power analysis confirmed that the study had excellent statistical power (>0.99) to detect the observed indirect effects.

**Table 3:** Association between post-traumatic growth and adherence to rehabilitation exercises (unadjusted).

Effect path	Effect size (*β*)	Boot SE	Boot LLCI	Boot ULCI	Proportion of total effect (%)
Total effect	0.586	0.044	0.495	0.665	
Direct effect	0.149	0.069	0.014	0.289	25.43
Total indirect effect	0.437	0.054	0.328	0.546	74.57
PTG-SE-REA	0.261	0.037	0.193	0.340	44.54
PTG-PSS-REA	0.176	0.047	0.087	0.271	30.03

PTG, post-traumatic growth; SE, self-efficacy; PSS, perceived social support; REA, adherence to rehabilitation exercises; Boot SE = bootstrap standard error; LLCI/ULCI = lower/upper limit of the 95% bias-corrected confidence interval based on 5,000 bootstrap samples.

**Table 4:** Association between post-traumatic growth and adherence to rehabilitation exercises (adjusted for income).

Effect path	Effect size (*β*)	Boot SE	Boot LLCI	Boot ULCI	Proportion of total effect (%)
Total effect	0.571	0.046	0.476	0.656	
Direct effect	0.149	0.069	0.016	0.290	26.09
Total indirect effect	0.422	0.046	0.316	0.524	73.91
PTG → SE → REA	0.249	0.038	0.181	0.330	43.61
PTG → PSS → REA	0.173	0.046	0.086	0.264	30.30

PTG, post-traumatic growth; SE, self-efficacy; PSS, perceived social support; REA, adherence to rehabilitation exercises; Boot SE = bootstrap standard error; LLCI/ULCI = lower/upper limit of the 95% bias-corrected confidence interval based on 5,000 bootstrap samples.

### 3.5 Testing for gender invariance in structural equation models

Furthermore, a sequence of multi-group analyses was performed to examine cross-gender measurement invariance. The Model A showed excellent fit (*χ*^2^ = 359.173, CFI = 0.964, TLI = 0.955, RMSEA = 0.054), providing a valid baseline. Imposing increasingly strict constraints, from Model B through Model D, did not significantly worsen model fit [all ΔCFI < 0.010, ΔRMSEA < 0.015, and *p*(Δχ^2^) > 0.05] (Lasker, 2024; Protzko, 2024), as shown in Table 5. Although Model E yielded a significant chi-square difference [Δ*χ*^2^(35) = 71.438, *p* < 0.01], the change in CFI remained within the acceptable threshold (ΔCFI < 0.010), and the absolute fit indices remained good (CFI = 0.958, TLI = 0.956, RMSEA = 0.053). Measurement invariance tests of the income-adjusted model were supported, with no significant fit deterioration from Models B to E (Table 6). Overall, the results support substantial measurement invariance across gender groups.

**Table 5:** Measurement equivalence tests for different gender cohorts (unadjusted).

Model	*χ* ^2^	df	χ2/df	CFI	TLI	RMSEA	Δχ^2^	∆df	*P* (Δχ^2^)	ΔCFI	ΔTLI	ΔRMSEA
A	359.173	165	2.177	0.964	0.955	0.054						
B	372.487	176	2.116	0.964	0.957	0.053	13.314	11	0.273	0	−0.002	−0.001
C	372.648	177	2.105	0.964	0.958	0.052	13.475	12	0.335	0	−0.003	−0.002
D	373.918	180	2.077	0.965	0.959	0.052	14.745	15	0.470	0.001	−0.004	−0.002
E	430.611	200	2.153	0.958	0.956	0.053	71.438	35	<0.01	−0.006	−0.001	−0.001

A = unconstrained, B = measurement weights, C = structural covariances, D = structural residuals, E = measurement residuals.

**Table 6:** Measurement equivalence tests for different gender cohorts (adjusted for income).

Model	χ^2^	df	χ^2^/df	CFI	TLI	RMSEA	Δχ2	∆df	P (Δχ^2^)	ΔCFI	ΔTLI	ΔRMSEA
A	417.950	187	2.235	0.969	0.960	0.047						
B	424.535	198	2.144	0.970	0.963	0.046	6.585	11	0.832	0.001	0.003	−0.001
C	425.249	204	2.085	0.970	0.965	0.044	7.299	17	0.979	0.001	0.005	−0.003
D	426.149	207	2.059	0.971	0.966	0.044	8.199	20	0.990	0.002	0.006	−0.003
E	456.299	227	2.010	0.969	0.968	0.043	38.349	40	0.545	0.000	0.008	−0.004

A = unconstrained, B = measurement weights, C = structural covariances, D = structural residuals, E = measurement residuals.

## 4 Discussion

The study found that PTG was associated with REA and may also be linked to it through the mediating roles of SE and PSS. This dual-mediation model offers a novel perspective on how positive psychological changes following trauma can translate into sustained healthy behaviors.

The findings indicate that patients with lower limb fractures exhibit moderate to high PTG levels (46.02 ± 12.18), consistent with previous research (Chen et al., 2025). Notably, PTG was positively associated with REA, accounting for 25.43% of the total effect. Within the framework of PMT, fractures are viewed as health threats, a cognitive appraisal that may be linked to adaptive behaviors such as REA (Steiner et al., 2021). Trauma survivors often experience PTG through positive cognitive restructuring, viewing trauma as an opportunity for growth, which fosters continued engagement in rehabilitation (Ntoumanis et al., 2021). Higher levels of PTG could also be related to greater patient autonomy in setting rehabilitation goals and participating in exercises (Flannery, 2017).

SE serves as a critical mediator between PTG and REA, with an indirect effect of 0.261, representing 44.54% of the total effect. This underscores the significance of patients’ beliefs in their ability to perform rehabilitation tasks. Self-efficacy is linked to behavioral execution, effort investment, and persistence, which are in turn associated with health behaviors (Southwell et al., 2022; Fuchs et al., 2025). Moreover, dimensions of PTG, like “individual strength” and “new possibilities,” may boost patients’ confidence in overcoming challenges, translating into compliance behaviors (Fu et al., 2022).

PSS serves as a mediator in the relationship between PTG and REA, exhibiting an indirect effect of 0.176, which corresponds to 30.03% of the overall effect. PMT emphasizes that threat appraisal includes recognizing external rewards, reflecting patients’ acknowledgment of support from others (Taheri-Kharameh et al., 2020). Research indicates that those who perceive strong social support tend to be more inclined to adopt effective coping strategies (Onyango et al., 2022; Zare et al., 2020). Furthermore, the PTG dimension of “relating to others” is positively correlated with patients’ perceived capacity to seek social resources and with lower feelings of loneliness, both of which are associated with better adherence (Jones et al., 2020).

While PTG exhibits a relatively small direct effect on REA (*β* = 0.149), its clinical significance and theoretical importance emerge within a complete mediation model. PTG showed a stronger total association with REA (*β* = 0.586), which was largely accounted for by the indirect pathways through SE and PSS. These findings support a theoretical shift in intervention focus, from viewing PTG as a standalone outcome to considering it as an upstream, facilitative factor (Tedeschi et al., 2025). Future psychosocial programs could initially foster PTG to subsequently channel the associated personal and relational resources into adherence behaviors through skill-building and support mobilization. Such a sequential, mechanism-informed approach might be associated with more sustainable improvements in adherence (Nigg et al., 2019) compared with interventions focusing solely on behavior.

The multigroup analysis supported the measurement invariance of the structural model across different genders, suggesting that the pathways linking PTG, SE, PSS, and REA function similarly for both male and female patients. This consistency suggests that interventions focusing on these psychological factors may have broad applicability, aligning with prior research (Poudel et al., 2020). Future studies could examine other potential moderators, such as age and cultural context, to further personalize rehabilitation approaches.

These findings advocate for integrated, psychologically-informed rehabilitation approaches. A potential “dual-track” intervention program could be explored: initially enhancing SE through phased rehabilitation goals and virtual reality training (Chao et al., 2024), followed by expanding social support networks via family involvement and online patient support platforms (Hassanzadeh et al., 2024; Wen et al., 2025). Additionally, interventions should be tailored to individual patient characteristics, focusing on SE improvement for introverted patients and supporting those lacking social resources (Liu et al., 2024). This approach may be positively associated with rehabilitation outcomes.

## 5 Strengths and limitations

The strengths lie in the integration of PTG within the PMT framework, which offers a novel theoretical contribution. Additionally, testing a parallel mediation model with gender invariance analysis provides robust and nuanced insights into the potential mechanisms influencing rehabilitation behavior. However, this research recognizes several limitations. First, the sample data were collected from a single research facility, which limited generalizability. Secondly, the cross-sectional design fundamentally limits causal inferences regarding the relationships between PTG, SE, PSS, and REA, and cannot elucidate their longitudinal dynamics throughout the rehabilitation process. Finally, future research could enhance the models by incorporating additional variables, such as coping styles, personality traits, and pain catastrophizing, to develop a deeper and more thorough understanding of the underlying mechanisms of influence.

## 6 Conclusion

The association of post-traumatic growth with adherence to rehabilitation exercises is mediated by self-efficacy and perceived social support. Interventions aimed at boosting post-traumatic growth, self-efficacy, and social support perception appear to be linked to more positive rehabilitation outcomes.

## Data Availability

The datasets presented in this article are not readily available because. The data that support the findings of this study are available from the corresponding author upon reasonable request. Requests to access the datasets should be directed to zengjieye@qq.com.
